# EMG-driven control in lower limb prostheses: a topic-based systematic review

**DOI:** 10.1186/s12984-022-01019-1

**Published:** 2022-05-07

**Authors:** Andrea Cimolato, Josephus J. M. Driessen, Leonardo S. Mattos, Elena De Momi, Matteo Laffranchi, Lorenzo De Michieli

**Affiliations:** 1 Rehab Technologies Lab, Fondazione Istituto Italiano di Tecnologia, Via Morego, 30, 16163 Genova, Italy; 2 Department of Electronics, Information and Bioengineering (DEIB), Neuroengineering and Medical Robotics Laboratory, Politecnico di Milano, Building 32.2, Via Giuseppe Colombo, 20133 Milan, Italy; 3 Department of Advanced Robotics, Fondazione Istituto Italiano di Tecnologia, Via Morego, 30, 16163 Genova, Italy

**Keywords:** Electromyograhy, Microprocessored-controlled lower limb prosthesis, Legged locomotion, Neuro-control

## Abstract

**Background:**

The inability of users to directly and intuitively control their state-of-the-art commercial prosthesis contributes to a low device acceptance rate. Since Electromyography (EMG)-based control has the potential to address those inabilities, research has flourished on investigating its incorporation in microprocessor-controlled lower limb prostheses (MLLPs). However, despite the proposed benefits of doing so, there is no clear explanation regarding the absence of a commercial product, in contrast to their upper limb counterparts.

**Objective and methodologies:**

This manuscript aims to provide a comparative overview of EMG-driven control methods for MLLPs, to identify their prospects and limitations, and to formulate suggestions on future research and development. This is done by systematically reviewing academical studies on EMG MLLPs. In particular, this review is structured by considering four major topics: (1) type of neuro-control, which discusses methods that allow the nervous system to control prosthetic devices through the muscles; (2) type of EMG-driven controllers, which defines the different classes of EMG controllers proposed in the literature; (3) type of neural input and processing, which describes how EMG-driven controllers are implemented; (4) type of performance assessment, which reports the performance of the current state of the art controllers.

**Results and conclusions:**

The obtained results show that the lack of quantitative and standardized measures hinders the possibility to analytically compare the performances of different EMG-driven controllers. In relation to this issue, the real efficacy of EMG-driven controllers for MLLPs have yet to be validated. Nevertheless, in anticipation of the development of a standardized approach for validating EMG MLLPs, the literature suggests that combining multiple neuro-controller types has the potential to develop a more seamless and reliable EMG-driven control. This solution has the promise to retain the high performance of the currently employed non-EMG-driven controllers for rhythmic activities such as walking, whilst improving the performance of volitional activities such as task switching or non-repetitive movements. Although EMG-driven controllers suffer from many drawbacks, such as high sensitivity to noise, recent progress in invasive neural interfaces for prosthetic control (bionics) will allow to build a more reliable connection between the user and the MLLPs. Therefore, advancements in powered MLLPs with integrated EMG-driven control have the potential to strongly reduce the effects of psychosomatic conditions and musculoskeletal degenerative pathologies that are currently affecting lower limb amputees.

## Introduction

### Microprocessor-controlled lower limb prostheses

Modern Microprocessor-controlled lower limb prostheses (MLLPs) represent a class of prosthetic devices that can simulate the joint’s biological behavior through real-time adaptive control driven by the sensory information acquired from embedded sensors (e.g. encoders, load and force cells) [42, 43]. Modern advancements in actuation and electronics have primarily led to the development of variable damping (passive) MLLPs and then to powered (active) MLLPs [131]. The main difference is that the former class of devices can only change the joint impedance, but unlike the latter, cannot actively generate net positive power. Compared to their fully passive (non-microprocessor controlled) predecessors, both types of devices can reproduce a broader repertoire of the human behaviour by restoring more dynamic functionalities.

In particular, variable damping lower limb prostheses guarantee the restoration of almost healthy-like locomotion of energy neutral or dissipating actions, such as walking, stair descent and sitting down. However, users have no direct control over the device [42]: it is the device itself that decides how to behave based on the sensors recorded information. This condition tends to lead to high cognitive fatigue and excessive energy consumption, especially during more complex activities [84]. User perception of inadequate controllability of the device, specifically a lack of intuitive control, reduces the acceptance rate of the lower limb prosthesis, which consequently leads to its abandonment [21, 108]. While comfort remains one of the key factors in prosthesis rejection [9, 99, 112], poor mobility is one of the leading causes of eventual device abandonment [44, 46, 104]. Additionally, users that do not abandon their prostheses incur high risks of developing a series of neuromusculoskeletal disorders and cardiovascular diseases [12, 45, 75, 92]. This is related to the inability of variable damping MLLPs to provide positive power: most types of basic healthy-like locomotion (e.g. walking) are governed by phases of positive power output [60, 88, 89, 95]. As a consequence, compensatory movements and gait asymmetry increase biomechanical stresses, particularly on the healthy biological joints of the amputee, causing articular pain to the knee, hip and back [8].

Active powered prostheses have been suggested to address the desire of being able to exert positive power during locomotion. In theory, these devices should allow for more natural gaits, and enable a wider range of possible movements and energy-generative actions, such as sloped gait, sit-to-stand, stair climbing and running [124]. However, their benefits have not yet been validated through biomechanical, performance-based and patient-reported metrics [42, 126]. Most likely, this validation stage has not yet been reached because of the poor user acceptance of these devices. Their poor acceptance can partly be explained by mechanical challenges that remain difficult to overcome. For example, the powered prostheses on the market are noisier, heavier, less smooth, and have shorter battery life than variable damping MLLPs. However, in addition to mechanical challenges, similarly to the variable damping prostheses, most powered prostheses do not provide direct control to the end-user: their control relies solely on interpreting embedded sensor data. Possibly, lack thereof is even more critical if the device allows for a wider range of activities.

### Neuro-control architecture

Concurrently, trends in prosthetics, orthotics and Human–Robot Interaction (HRI) are pushing not only for the restoration of essential human locomotion, but also for the user to have direct control to the device through the neural pathways [52]. Neuro-controllers in fact have the capabilities to decode the neural activity either from the central or the peripheral nervous system in order to control external devices. In particular, it is possible to use EMG sensors to measure and decode users’ motion intention directly from neural activity using muscles as terminal amplifiers of motor afferent commands [94].

Human locomotion neuro-control comprises two particular sets of commands: *volitional* and *rhythmical*. Rhythmic locomotion occurs when humans use repetitive limb movements to translate in space, such as walking and running. Usually, this class of motor patterns does not involve conscious intervention on behalf of the subject, but they result from sensory-motor reflexes activated from specific neural networks [81]. Scientific evidence locates these so-called Central-Pattern Generators (CPGs) in the spinal cord or in the brain stem and they trigger organized muscle contraction in a cyclic manner [2, 30]. Their activation and regulation can both derive from Central Nervous System (CNS) inputs and sensory-motor feedback [31, 82]. Volitional movements instead are a broad class of movements that involve motion planning and motor control, like non weight-bearing knee flexing. Conscious movements require constant and vigilant attention from the CNS involving different cortex areas and intensive collaboration across the diencephalon, brainstem, and cerebellum [73].

**Fig. 1:**
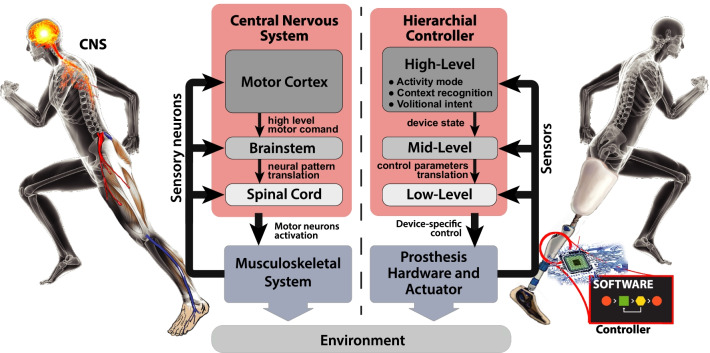
Hierarchical controller schematic representation. Comparison between a generalized control framework for microprocessor-controlled lower limb prostheses (right) and the human motor control (left). The figure displays the classic interactions between the Hierarchical Controller layers and user-device-environment. Controller level-specific tasks are listed in the figure (adapted from [124])

To achieve the same locomotion control, MLLPs have different levels in their control architecture that is structured similarly to the human neuro-control system. This particular multi-layer control framework is referred to as a hierarchical controller, and shown in Fig. 1 [124]. In particular, the high-level control is tasked with identifying and quantifying the user’s intention. This primarily regards selection of user *activity mode* (e.g. walking, slope ascending or sitting [111]) and, secondarily, *context recognition* (e.g. ambulation speed and swing or stance phase [119]). The more these selections stem from user awareness and volition, the more difficult it is to identify user volition based only on embedded sensors, such as joint encoders, load cells and an Inertial Measurement Units (IMU). However, the use of EMG sensors is a valid choice for the reliable identification of *volitional intent*, which paves the way for direct neuro-control of the device [56].

The identified intent from the high-level controller is fed to the mid-level controller, which translates it to *device state control*, such as a target joint impedance, stiffness or angular trajectories. Finally, the *device-specific controller* in the low level interprets the output of the mid-level controller, and closes the loop with the device-specific physical hardware, e.g. by controlling motor currents. The mid- and low-level controls are well comparable to the human’s brainstem and spinal cord, which—in contrast to the high-level control—can function well by relying solely on the aforementioned system dynamics without requiring much input or intervention of the human’s awareness and volition.

### Scope of the review

Among different neuro-controllers, roughly 80% of the papers collected on lower limb prosthesis control published after 2010 are related to EMG-driven control [131], which indicates the recent popularity of this subject. This particular research interest in myoelectric control—also known as EMG-driven control—is related to the necessity of novel technological solutions to support the development of new powered lower limb prostheses [50, 52, 131]. Myoelectric control represents a possible candidate for introducing a user’s direct control for powered MLLPs. Although the literature promotes this technology and its positive benefits for the end user, such as reduction of phantom limb pain [80, 96], it is not mature enough for powered MLLPs. In fact, no commercial device is available with such technology, unlike their upper limb counterparts.

Only one comprehensive analysis has been found that focuses on EMG-driven control in lower limb prostheses [41]. Whereas various reviews have been published on MLLPs, most of these focus on mechanics and control, and either do not discuss EMG-driven control [50, 52, 69], or dedicate a short section to it [42, 84, 124, 131]. Instead, whereas numerous other reviews focus specifically on myoelectric control, they either do so for generic HRI [1, 3, 93, 94, 97, 103, 109], or for upper limb prosthetics specifically [47, 102, 107].

In the attempt to differentiate from previous reviews, we are focusing our survey on the novel and more challenging implementations of EMG-driven control in high-level rather than mid- or low-level control. Information and discussions about mid- and low-level control can be found in [52, 69, 124, 131]. Therefore, this work aims at providing a detailed and organized systematic overview of myoelectric control for powered MLLPs prostheses. The main objective of this review is to analyze the merits and drawbacks of the various implemented EMG-driven controllers. To do so, we defined four major topics of investigation categorizing the most important and common characteristics in the available literature. This analysis was conducted to provide an unbiased and systematic evaluation of the literature on EMG-MLLPs. In addition, the goal is to discriminate the potentialities and limitations of each technological embodiment to define a clear path for future research investment.

## Methods

### Eligibility criteria

The first eligibility criteria for this review was the actual dissertation of a EMG-driven control for MLLPs. Papers were considered only if presenting: (1) an implementation of the myoelectric controller, (2) a complete description of its architecture, and (3) results on a physical or simulated device.

Since EMG-driven control for MLLPs represents a niche research sector in both HRI and prosthetics, authors decided to not include any limitation in the year of publication, type of publication (e.g., journal or conference papers) and language.

### Information sources, search strategy and selection process

Searches have been conducted in several major scientific research databases. The following databases were chosen based on the number of results obtained with the search phrase “myoelectric control lower limb” in Google Scholar: PubMed, ScienceDirect, IEEE Xplore, Springer Link, ASME, NCBI and ResearchGate. Successively, combinations of the words “myoelectric”, “EMG”, “control”, “lower limb”, “knee”, “ankle” and “prosthesis” were used for the actual search in the cited databases. Results were not screened based on the date of publication and type of publication. Databases were last consulted for this review in May 2021.

Due to high prevalence in the results of papers related to upper limb prostheses, orthoses and other robotic devices, and other papers not related to EMG-driven lower limb prosthesis controllers, a manual selection was made. Only papers regarding knee and ankle prosthesis controllers driven from EMG acquisitions were included based on title and abstract. Content of the remaining articles was reviewed. Papers were included in the review process if they presented an actual implementation of the myoelectric controller, a complete description of its architecture, and results on a physical or simulated device. Finally, all cited papers from the manuscripts obtained from the screening strategy were collected and went through the same search and selection process.

**Fig. 2:**
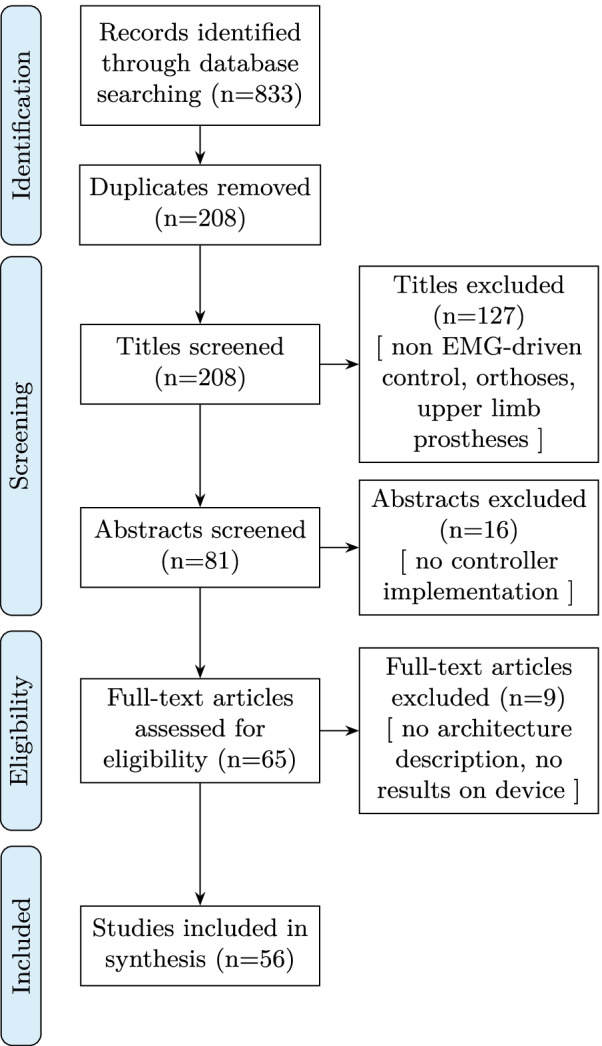
Systematic review flow diagram. The PRISMA flow diagram for the systematic review, detailing the database searches, the number of abstracts screened and the full texts retrieved

The diagram in Fig. 2 illustrates the number of manuscripts that were identified, screened and finally included in this study.

### Data items: topics of investigation

The data collected from this review is divided according to four major topics used to structure the literature investigation:

- Neuro-control: This aspect discusses how the nervous system, through the muscles, can control the lower limb prosthesis. It concerns the use of input neural signals (i.e. EMG) for the generated output movement, rather than the specific implementation of controllers. In particular, it explores the types of movement that can be restored, namely either rhythmic locomotion or volitional movement. This is consequently reflected on the implementation of different EMG-driven control strategies: Computational Intrinsic Control (CIC) or Interactive Extrinsic Control (IEC) [84].
- EMG-driven working principles: The methodologies employed in EMG-driven controllers to translate user intention and volition to high-level control parameters have been group in three major categories: direct control, pattern recognition, and model-based. Each of this class adopt a different principle to interpret user volition and intention from EMG signals and translate it in to a high-level control.
- Neural input and processing: Depending on the type of MLLP, the level of amputation and control class, and the choice of muscles, different sensors and processing methods can be applied. Identifying these differences or similarities is essential to confront methodologies in relation with the final results.
- Performance assessment: This topic discusses the collection of meaningful reported results in the reviewed literature to provide the reader an overview of the achieved performance of the controllers.

### Data collection process, study of risk bias assessment, effect measures

The first author defined the main source databases and conducted the search and selection process. No risk bias has been identified for the data selection process. However, to avoid possible apophenia and contextual biases in the collection process, the second author revised the proposed data items and defined detailed subcategories for each item (topic of investigation). For each subcategory, the first author chose effect measures that were recurrent among all reviewed manuscripts. Effect measures are collected in different form depending from the topic of investigation. The rest of the authors inspected the synthesis results to ensure no incongruity among the collected measures and the data clustering.

### Synthesis methods

The authors chose to synthesize the collected data in a tabular form: one table for each investigated topic. Data was catalogued in sub-fields representing relevant information for the implementation and evaluation of the EMG-driven controller in MLLPs. In each table, all the collected papers are cited and grouped by the type of working principle. Paper citations for each working principle are ordered by date of publication. If manuscripts employ identical concepts, they are grouped in a single table entry and cited collectively.

**Fig. 3:**
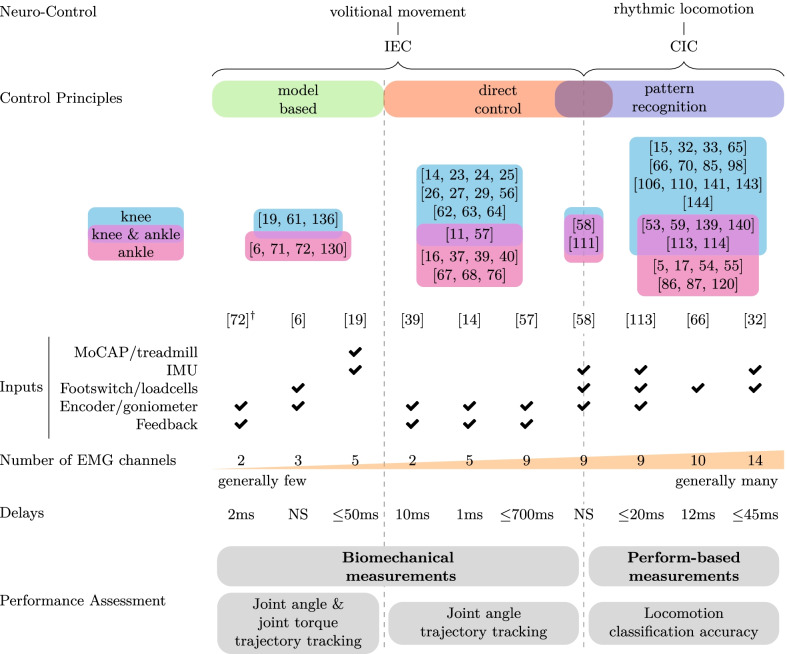
Graphical overview of the reviewed literature, grouped by the same four topics used in the manuscript. The references are grouped by application (knee, ankle or both), the EMG-driven control working principle and type of neuro-control (type of movement restored). Some of the most relevant works are detailed additionally by listing the employed additional inputs, number of EMG channels and controller delays, if known. Five classes of additional data inputs were recognized (apart from EMG signals). Motion Capture (MoCap) and sensorized treadmills with force platforms are used to acquire body kinematic and dynamic data; IMU sensors are usually integrated to obtain orientation of the lower limb segments; footswitch and loadcells are installed to acquire the force exchanged with the environment; encoders and goniometers are used to measure joint angles; finally, visual or haptic feedback is sometimes provided to the user to encourage a correct employment of the device. On the lower part of the graph, the most used measurements for each control class validation is displayed. *NS* not stated; ^†^referenced work belong to a hybrid model-based and pattern recognition control class, see “EMG-driven working principles” section

In addition, a graphical diagram was designed to navigate the collected data in the tables and to show the possible interconnections between the four topics of investigation (Fig. 3). The diagram organizes and synthesizes the collected literature according to the obtained data items. Some of the most representative papers were moreover used as examples for better displaying the obtained results.

## Results

This section presents the complete listing of the review findings, divided in the four topics of investigation as described in “Data items: topics of investigation” section.

In order to aid the reader in navigating the results and to give a better understanding of the interconnection of these four topics, a graphical overview of the reviewed literature is presented in Fig. 3. From this graph it is possible to advance some preliminary results. For example, looking at neuro-control, the number of works that investigate the control of prosthetics in volitional movement (IEC) and rhythmic locomotion (CIC) is balanced (29 papers each), but pattern recognition controllers are more numerous in literature. It can be additionally noticed that there is no particular preference regarding the control type with respect of the type of prosthetic joint. A similar result has been found for the type of additional sensors, when inspecting the possible control signal inputs. Instead, works that employ pattern recognition principles generally use more EMG channels ($6.6\pm 3.1$ channels on average) than those employing direct control and model-based control principles (respectively $3.2\pm 1.9$ and $2.5\pm 1.1$ channels on average). Volitional movement controllers use generically more often visual/haptic feedback (almost 50% of the collected papers). Looking at the performance assessment, no significant pattern can be determined on the control delay, which refers to the time between muscle excitement and control execution. It is anyway interesting to notice that two out of ten of the selected papers did not report any information about this parameter, while being an important metric to evaluate the HRI. Finally, the diagram shows a strong preferences for performing biomechanical evaluations (joint angle and torque trajectories) on IEC controllers, while perform-based measures (locomotion classification accuracy) are preferred instead on CIC.

Specific results of each of the four categories and their interconnections are listed separately in the following sections.

### Neuro-control

Results on the neuro-control abilities are collected in Table 1.

**Table 1:** Overview of the neuro-control capabilities of the device

Ref.	Control strategy	Neuro-control	Actuator control signal	Joint	Platform
[64]	IEC	Direct control on the joint lock mechanism	Switch signal of the electromagnetic clutch	Knee	E.C.P. (Electro-Control Prosthesis)$^{{\star } {\dag }}$
[29]	IEC	Voluntary control of joint FE	Servo-amplifier electrohydraulic valve level	Knee	Prosthesis simulator (hydraulic system externally supplied and controlled)$^{{\star } {\dag }}$
[24–27]	IEC	Voluntary control of joint FE	Joint angle reference	Knee	ABS$^{\star }$; off-line VS$^{\dag }$
[56]	IEC	Voluntary control of joint FE	Joint torque reference	Knee	Vanderbilt micro-controlled leg prosthesis$^{{\star } {\dag }}$
[14, 23, 62, 63]	IEC	Voluntary control of joint FE	Joint torque reference	Knee	Clarkson university knee powered prosthesis prototype$^{{\star } {\dag }}$
[57]	IEC	Voluntary control of joint FE and IE	Joint torque reference	Knee, ankle	Virtual environment$^{\star }$, powered knee prosthesis prototype (Center for Bionic Medicine, Rehabilitation Institute of Chicago)$^{\dag }$
[37]	IEC	Direct control on joint angle movement	Joint angle reference	Ankle	Passive prosthetic feet$^{\star }$; on-line VS$^{\dag }$
[16]	IEC	Voluntary control of joint FE	Joint angular velocity	Ankle	On-line VS$^{{\star } {\dag }}$
[67, 68]	IEC	Voluntary control of joint FE	Force reference of artificial pneumatic muscles	Ankle	Artificial pneumatic muscles powered ankle prosthesis prototype (University of Michigan)$^{{\star } {\dag }}$
[76]	IEC	Voluntary control of joint FE	Joint angle reference	Ankle	On-line VS$^{{\star } {\dag }}$; ankle prototype $^{\dag }$
[11]	IEC	Voluntary control of joint FE	Joint angle reference	Knee, ankle	ABS$^{\star }$; off-line VS$^{\dag }$
[39, 40]	IEC	Voluntary control of joint FE	Joint torque reference	Ankle	On-line VS$^{{\star } {\dag }}$
[98]	CIC	Control of walking control ground-level or slopes	NI	Knee	Four-bar linkage mechanism, Ottobock$^{\star }$; Endolite, Blatchford$^{\star }$
[70]	CIC	Adaptive control based on locomotion recognition	Stepper motor control driving a gear train	Knee	Prototype leg prosthesis (step motor driving the shaft of six-bar knee)$^{{\star } {\dag }}$
[5]	CIC	Transition between level-ground to stairs intrinsic adaptive control	Joint position/torque (control state dependent)	Ankle	On-line VS$^{\star }$; BiOM ankle-foot prosthesis, MIT Media Lab$^{\star }$
[65, 66, 141, 144]	CIC	Adaptive control based on locomotion recognition	NI	Knee	Mauch SNS, Össur$^{\star }$; off-line VS$^{\dag }$
[66]	CIC	Adaptive control based on locomotion recognition	NI	Knee	Hydraulic passive knee$^{\star }$; off-line VS$^{\dag }$
[53]	CIC	Adaptive control based on locomotion recognition	Position and velocity joint trajectory	Knee, ankle	NS$^{\star }$; off-line VS$^{\dag }$
[85]	CIC	Adaptive control based on walking phase recognition	NI	Knee	ABS$^{\star }$; off-line VS$^{\dag }$
[15, 110]	CIC	Adaptive control based on walking phase recognition	NI	Knee	ABS$^{\star }$; off-line VS$^{\dag }$
[32, 33, 143]	CIC	Adaptive control based on locomotion recognition	NI in passive MLLPs; joint torque for active MLLP	Knee	Knee–ankle powered prototype$^{{\star } {\dag }}$
[86, 87]	CIC	Adaptive control based on locomotion recognition	NI	Ankle	Passive ankle$^{\star }$; off-line VS$^{\dag }$
[120]	CIC	Joint DoF motion determination	NS	Ankle	On-line VS$^{{\star } {\dag }}$
[58, 111]	CIC-IEC	Adaptive control based on locomotion recognition; non-weight bearing voluntary control of joints FE	Joint torque reference	Knee, ankle	Vanderbilt micro-controlled leg prosthesis$^{{\star } {\dag }}$
[59, 113, 114, 139, 140]	CIC	Adaptive control based on locomotion recognition	Joint torque reference	Knee, ankle	Vanderbilt micro-controlled leg prosthesis$^{{\star } {\dag }}$
[17]	CIC	Adaptive control based on terrain slope estimation	Joint damping reference	Ankle	Peking university PKU-RoboTPro$^{{\star } {\dag }}$
[106]	CIC	EMG-triggered stride motion routine	Motor current reference	Knee	Prototype leg prosthesis$^{{\star } {\dag }}$
[54, 55]	CIC	Adaptive control based on locomotion recognition	NI	Ankle	ABS$^{\star }$; off-line VS$^{\dag }$
[6]	IEC	Voluntary control of joint FE	Joint angle reference	Ankle	On-line VS$^{{\star } {\dag }}$
[61]	IEC	Voluntary control of joint FE	Joint torque reference	Knee	ABS with ABA and powered knee prosthetic prototype$^{{\star } {\dag }}$
[136]	IEC	Voluntary control of joint FE	Joint torque reference	Knee	ABS with ABA and Vanderbilt micro-controlled leg prosthesis$^{{\star } {\dag }}$
[71, 72, 130]	CIC-IEC	Voluntary control of joint FE	Joint torque reference	Ankle	BiOM ankle-foot prosthesis, MIT Media Lab$^{{\star } {\dag }}$
[19]	IEC	Voluntary control of joint FE	Joint torque reference	Knee	ABS$^{\star }$; on-line VS$^{\dag }$

Fields include: paper reference; control strategy (the neural control strategy used for the high-level control function implementation: CIC or IEC); neuro-control (the use of input neural signals for the generated output movement); actuator control signal (the output signal from the high-level EMG-driven control); joint (the controlled lower limb joint); platform (the device used for acquisition and testing)

*NI* not implemented, *NS* not stated, *IEC* interactive extrinsic contro, *CIC* computational intrinsic contro, *FE* flexion-extension; *IE* internal-external rotation, *DoF* Degrees of Freedom, *ABS* able-bodied subjects, *ABA* able-body adaptor, *VS* virtual simulator

$^{\star }$Platform for data acquisition

$^{\dag }$Platform for control testing

**Fig. 4:**
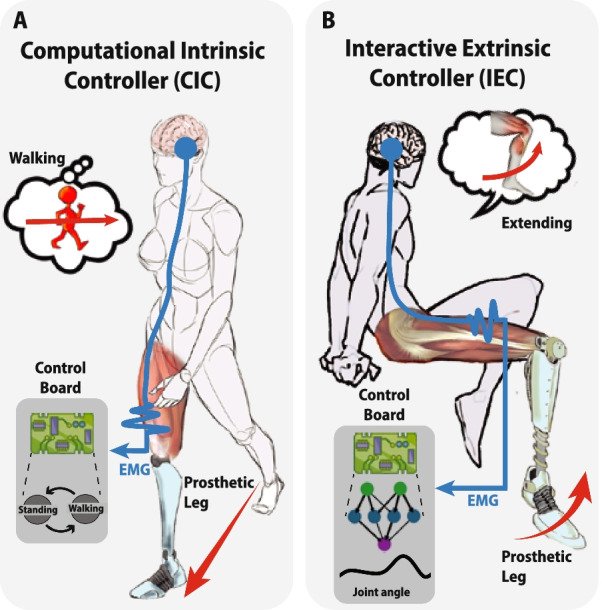
Computational Intrinsic Control (CIC) and Interactive extrinsic Control (IEC) approaches for lower limb prosthesis neuro-control. Comparison between CIC (on the left) and IEC (on the right) from motor task generation to EMG-driven control. **A** CICs chose the correct control among the states implemented in the control board based on the EMG signals generated during a rhythmic locomotion; **B** IECs instead transform EMG recorded patterns to a specific continue modulation of the prosthetic joint

Martin et al. categorized rhythmical and volitional type of movements in two general behaviours that a high-level controller can implement: CIC and IEC [84]. These essentially distinguish between controllers that are based on the conscious involvement of the user (volitional or IEC) and those that are not (rythmical or CIC) (Fig. 4). Correspondingly, the adoption of this particular subdivision allows us to classify the EMG-driven controllers based on how the neural information is employed (Fig. 3).

CICs typically decode the user’s motor intention and prosthetic device state through data from embedded neural sensors This information is then employed to establish an appropriate control in order to accommodate for the changes in the locomotion [121]. This is observable in Table 1: the majority of CICs are characterized by a neural-control based on adaptive controls depending on locomotion recognition. CICs represent the state-of-the-art in the control of non-EMG MLLPs and they have been explored with different approaches, both in the literature and in commercially available devices [42].

Instead, IECs are designed to guarantee continuous communication between the user and the device so as to directly modulate the prosthesis’ state (Table 1). In fact, this type of control, differently from CIC, cannot be implemented in non-EMG MLLPs since no neural input is provided to the prosthetic device. As such, IECs have enabled the control of lower limb movements that were not possible with CIC.

From the obtained results, no particular correlation among the remaining fields was found. This suggests that there is no particular influence of the choice of neuro-control on the specific prosthetic device, but only on the prioritized type of movement to be restored.

### EMG-driven working principles

Table 2 provides an overview of the control strategies and working principles of the reviewed EMG-driven approaches for MLLPs. The table also shows that the reviewed literature has been classified according to the following three types of control: Direct Control, Pattern Recognition Control and Model-Based Control.

**Table 2:** Overview of lower limb EMG-driven controllers working principles

Ref.	Walking controller	Slope/speed adaptation	Additional modalities	Training/calibration time
*Direct control*				
[64]	EMG-triggered knee joint lock during stance phase	SlA$^{\star }$, SpA$^{\star }$	All (STA$^{\star }$)	NN
[29]	EMG-proportional modulation of knee joint velocity	SlA, SpA$^{\star }$	All (not tested)	NS
[24–27]	ML-driven knee joint angle trajectory generation	SlA, SpA	All (not tested)	CT: 10–15 s, per 2 sessions, per 5 days
[56]	EMG-driven knee joint stiffness set-point	SlA, SpA	All (NWB$^{\star }$)	ST: 1 h, before each use
[14, 23, 62, 63]	EMG-driven knee joint stiffness set-point	SlA, SpA	All (STND$^{\star }$, SIT$^{\star }$, SQ$^{\star }$, STA$^{\star }$, NWB$^{\star }$)	ST: 3 h, per 4 sessions; CT: 2 h trajectory tracking trials
[57]	EMG-driven multi-DoF knee and ankle joint stiffness set-point	SlA, SpA	All (NWB$^{\star }$)	ST: therapist session; CT: 3 s per 64 trials, per 4 sessions
[37]	ML-driven knee joint angle trajectory generation	SlA, SpA	All (not tested)	NS
[16]	EMG-driven ankle joint stiffness set-point	SlA, SpA	All (NWB$^{\star }$)	CT: 10 trials ($\sim$ 80 s)
[67, 68]	EMG-proportional plantarflexor torque generation	SlA, SpA	All (not tested)	CT: NS
[76]	EMG-triggered ankle plantarflexion and dorsiflexion	NI	NI	CT: NS
[11]	EMG-decoded ankle and knee joint angle trajectory generation	SlA$^{\star }$	All (STA$^{\star }$, STD$^{\star }$)	CT: $\sim$ 20 trials per task
[39, 40]	EMG-proportional plantarflexor torque generation	SlA, SpA	All	ST: limited acclimation period
*Pattern recognition control*				
[98]	EMG-driven knee FSM (Stance [Post-HS, FF and Pre-TO], swing [SF, SE])	SlA	NI	Adaptation period of 20 min; FSM CT: NS
[70]	Knee joint moment control as function of EMG-driven locomotion identification	SlA$^{\star }$, SpA$^{\star }$	STA$^{\star }$, STD$^{\star }$	FSM CT: NS
[5]	EMG-driven FSM for level ground walking and stairs climbing	SlA, SpA	STA$^{\star }$	FSM CT: NS; ST < 20 min
[65, 66, 141, 144]	ML-driven knee joint FSM (Stance [Post-HS, Pre-TO], swing [Post-TO, Pre-HS])	SlA$^{\star }$	OBST$^{\star }$, STND$^{\star }$, STA$^{\star }$, STD$^{\star }$, TURN$^{\star }$	FSM CT: $\sim$ 15 min (3 times each task)
[53]	CPG-generated knee and ankle joint trajectories as function of ML-driven locomotion identification	NI	STND$^{\star }$, SIT$^{\star }$, STA$^{\star }$, STD$^{\star }$	FSM CT: NS
[85]	ML-driven knee joint FSM (Stance [Post-HS, FF, Pre-TO], swing [Post-TO, Pre-HS])	NI	STA$^{\star }$, STD$^{\star }$	FSM CT: 50 gait cycles per task
[15, 110]	ML-driven knee joint FSM (Stance [Post-HS, FF, Pre-TO], swing [Post-TO, Pre-HS])	NI	NI	FSM CT: 70 gait cycles
[32, 33, 143]	ML-driven knee joint FSM (Stance [Post-HS, Pre-TO], swing [Post-TO, Pre-HS])	SlA$^{\star }$	STA$^{\star }$, STD$^{\star }$	ST: therapist sessions; FSM CT: $\sim$ 30s (5 times per task)
[86, 87]	ML-driven ankle joint FSM (Stance [Post-HS, Pre-TO], swing)	SlA$^{\star }$, SpA$^{\star }$	STA$^{\star }$, STD$^{\star }$	FSM CT: 21 trials in total, 6–7 steps per trial
[120]	ML-driven FSM for multi-DoF ankle joint	SlA, SpA	All (NWB$^{\star }$)	FSM CT: 3 s per 8 trial, per 7 tasks
[58, 111]	ML-driven knee joint FSM (Stance [Post-HS, Pre-TO], swing [Post-TO, Pre-HS])	NI	STND$^{\star }$, SIT$^{\star }$, NWB$^{\star }$	FSM CT: NS
[59, 113, 114, 139, 140]	Knee and ankle joint impedance characterization as function of ML-driven locomotion identification	SlA$^{\star }$	STA$^{\star }$, STD$^{\star }$, SIT$^{\star }$, NWB$^{\star }$	Intrinsic controller parameters tuning (NS); FSM CT: 10–20 trials per task
[17]	ML-driven ankle joint impedance characterization based terrain inclination classification	SlA$^{\star }$	NI	Intrinsic controller parameters tuning (NS); CT: 3 sessions; ST: $\sim$ 5 h
[106]	EMG-triggered knee joint motion routine	NI	NI	NS
[54, 55]	ML-driven ankle joint FSM	SlA$^{\star }$	STA$^{\star }$, STD$^{\star }$	FSM CT: 5 gait cycles per trial; ST: 5 min per task
*Model-based control*				
[6]	EMG-driven model-based ankle joint angle trajectory generation	SlA, SpA	All (NWB$^{\star }$)	Virtual environment training: NS
[61]	EMG-driven model-based knee joint impedance characterization	SlA, SpA	All (not tested)	CT: NS
[135, 136]	EMG-driven model-based knee joint impedance characterization	SlA, SpA	All (NWB$^{\star }$)	CT: trajectory tracking trials, walking experiments
[71, 72, 130]	EMG-modulation of model-based ankle joint moment trajectory	SlA, SpA	All (STA$^{\star }$, STD$^{\star }$)	CT: 10 steps
[19]	Hybrid ML-NMS model-based knee joint moment generation	SlA, SpA$^{\star }$	All(STND$^{\star }$, SIT$^{\star }$)	CT: 3–10 trials per motor task

Fields include: paper reference; walking controller (the high-level control law during the walking cycle); slope/speed adaptation (the ability of the walking controller to adapt to different slope angles and ambulation velocities); additional modalities (additional types of locomotion supported from the EMG-driven controller); training/calibration time (required time to either calibrate the controller parameters or train the subject)

*NN* not necessary, *NS* not stated, *NI* not implemented, *ML* machine learning, *NMS* neuromuscularskeletal, *CPG* central pattern generator, *HS* heel strike, *FF* foot flat, *TO* toe off, *SF* swing flexion, *SE* swing extension, *FSM* finite-state machine, *DoF* Degrees of freedom, *SlA* slope adaptation, *SpA* speed adaptation, *All* no restriction in the locomotion control, *NWB* non-weight bearing joint movements joint movement, *STND* standing, *SIT* sitting, *SQ* squatting, *STA* stairs ascending, *STD* stairs descending, *OBST* obstacle stepping, *TURN* turning on the spot, *CT* calibration time, *ST* subject training

$$^{\star }$$Tested modalities

Independently from the type of controller, an essential feature is the walking control. Walking represents the most important movement in bipedal locomotion and is therefore essential to be restored in lower limb amputees. Consequently, the primary use of EMG signals is to accomplish healthy-like ambulation. The use of EMG signals and the design of the controller depends on the type of EMG-driven controller.

**Fig. 5:**
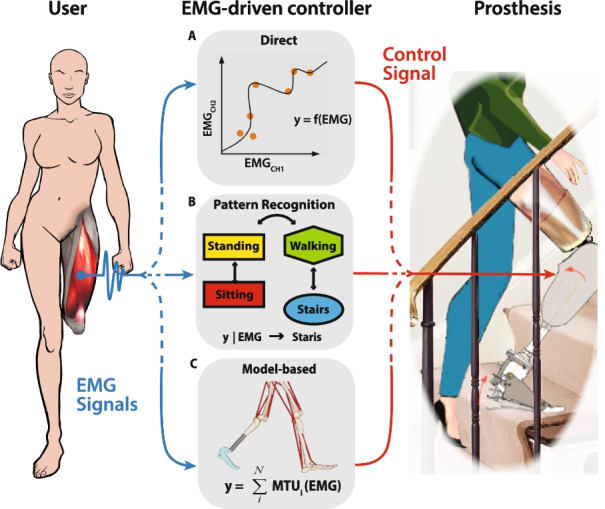
Graphical representation of the three possible solutions for EMG-driven control. EMG signals are acquired from the stump of the amputee and used as input for the high-level controller, depending on how the neural signals are used three type of EMG-driven controllers can be implemented: **A** direct control, **B** pattern recognition, **C** model-based; the control signal output is then used from the internal lower level control of the prosthetic device

Direct EMG-driven controllers refer to all those controllers that employ EMG as input of a specific function $y = f(x_{\text {emg}})$, where *y* represents the variables to be controlled during the walking cycle for the generation of the signal reference, such as joint angle or torque (Fig. 5a). Examples of these are proportional controllers [29, 64], regression functions [24, 37] or mapping-transformation functions between processed multi-channel EMG acquisitions and control parameters [56, 57, 62].

Pattern recognition control represents the largest class. They are reminiscent of (FSM)-based controllers, which are currently used in commercial MLLPs (Fig. 5b). FSM-based controllers distinguish a number of control states (actions) through state classification (perception), a concept similar to that governed by pattern recognition algorithms, a branch of Machine Learning (ML) [18]. Typical pattern recognition algorithms identify particular signals’ signatures (features) that can be observed under particular conditions. These features can be used, consequently, as transition rules in the controller to distinguish the different working conditions [70, 98, 106], such as slope adaptation (SlA) and speed adaptation (SpA). For this reason, as illustrated in Table 2, pattern recognition control algorithms provide only a pre-defined number of locomotion types with respect to the other controllers, such as only for stair ascend and descend, standing and sitting. Instead, direct controllers allow theoretically *all* types of motion.

The last class of EMG-driven controllers employ Musculoskeletal (MS) models to reproduce human kinematics and dynamics in locomotion [7, 83, 119] (Fig. 5c). Modeling in bio-engineering research has been used to study biological systems, whose characteristics are not directly measurable. In case of lower limb prostheses, it is possible to use MS to estimate joint impedance starting from the measured external forces and joint trajectories [34]. Additionally, with the use of EMG signals and translating them to appropriate muscles activation, these models can be used to modulate voluntarily tracked joint impedance [6, 61, 130, 136].

Another important distinction regarding the EMG-driven working principles is the different partitioning between user-training and control-calibration time. Both describe the time the user needs to spend with the prosthesis to achieve an effective Human-In-The- Loop (HITL) control of the device [79]. In fact, intra- and inter-subject variability of the biological system and of the signals involved in the control loop increase uncertainty in the control performance [132]. Stochastic variability within HITL can be reduced by tuning internal control variables, but it cannot be removed completely [133]. For this reason, the user has to be trained to avoid control errors and understand how to use the device optimally. As such, while calibration time is correlated to the number of tunable variables and provides an indication of control complexity, the training time is an indicator of how seamless and intuitive the control is. From the collected data (Table 2), it can be observed that the ratio between calibration and training time changes drastically in pattern recognition control. In fact, multiple recordings are necessary for the appropriate calibration of the FSM, but after this initial effort the user needs a minimal amount of training. The contrary can be said for the direct control: short initial parameter tuning, usually based on previous analysis, followed by prolonged training sessions.

The classification of EMG-driven control type is closely related to the neuro-control strategy since the analyzed literature shows that the pattern recognition algorithms are all CIC controllers, whereas direct control and model-based approaches are IECs. This can be observed also in Fig. 3. While the neuro-control distinction allows the reader to understand which are the target motor tasks intended to be restored, the working principle describes the methodology employed for the control.

### Neural input and processing

Table 3 lists the used muscles and applied signal processing techniques to provide input data to the EMG-driven controller.

**Table 3:** Overview of the used input signals and applied processing

Ref.	Muscles	Additional sensors and feedback	EMG signal processing (filter order and cut-off frequencies)	Window sampling	Classifier (features)
[64]	Single muscle not contracting during walking	NP	NI	Analog	Thresholding (Raw Sign.)
[29]	GRAC	Load cell on the knee pivot$^{{\star } {\dag }}$; footswitch$^{{\star } {\dag }}$;	RECT, BPF (NS)	Analog	NA (ENV)
[24–27]	Knee FLEX (RFEM/VASI/ VASL), knee EXT (SEMT)	Goniometer$^{\star }$	BPF (20–500 Hz)	115–192 ms	LM Network (HIST, ARC)
[56]	HAMS, QUAD	Joint encoder$^{\dag }$ (visual feedback of knee joint position)	HPF (1st ord, 20 Hz), RECT, LPF (1st ord, 2 Hz), NORM, PCA	2 ms	QDA (ENV)
[14, 23, 62, 63]	VASL, VASM, RFEM, SEMT, BICFL	Joint encoder$^{\dag }$ (visual and haptic feedback of knee joint position)	BPF (2nd ord, 20–450 Hz), RECT, LPF (2nd ord, 2.5 Hz), NORM; PCA	3 ms	NA (ENV)
[57]	SEMT, SAR, TFL, ADDL, GRAC, RFEM, VASM, VASL, BICFL	Joint encoder$^{\dag }$ (visual feedback of knee joint position)	NS	200 ms	LDA (MAV, NZC, SSC, WL)
[37]	GASM/SOL, TIBA	Joint encoder$^{\star }$	BPF (2nd ord, 10–500 Hz), RECT, LPF (2nd ord, 10 Hz)	50 ms	NARAX (ENV)
[16]	GASM, TIBA	NP	RECT, LPF (3rd ord, 2.5 Hz); PCA	10 ms	NA (MAV)
[67, 68]	GASM/GASL	MoCap system$^{\star }$; sensorized treadmill$^{\star }$; control signal visual feedback$^{{\star } {\dag }}$	FPF (2nd ord, 100 Hz), RECT, LPF (2nd ord, 4 Hz), NORM	NS	NA (ENV)
[76]	VASM, BICFL, TIBA, GASL	Ankle goniometer$^{\star }$, accelerometer$^{\star }$	RECT, LPF (NS)	NS	Peak detector (ENV)
[11]	VASL, RFEM, SEMT, BICFL	MoCap system $^{\star }$	NORM, BPF (4th ord, 30–350 Hz), RECT, LPF (4th ord, 6 Hz), Kalman filter	< 500 ms	NA (ENV)
[39, 40]	TIBA, GASM	Joint position$^{{\star } {\dag }}$ (visual feedback)	HPF (2nd ord, 20 Hz), RECT, LPF (2nd ord, 2 Hz)	10 ms	NA (ENV)
[98]	GLMAX, GLMED, TFL	Footswitch$^{{\star } {\dag }}$	LPF (2nd ord, 1 KHz), HPF (3rd ord, 50 Hz), NORM	NS	Thresholding (ENV)
[70]	VASL, VASM, RFEM, TFL, ADDL, BICFL, SEMM, SEMT	Footswitch$^{\star }$; load cell$^{\star }$; MoCap system$^{\star }$	NS	NS	Heuristic Tree (IDE, MAV, MDF, MF)
[5]	TIBA, GASM, GASL	Footswitch$^{{\star } {\dag }}$; potentiometer$^{{\star } {\dag }}$; encoder$^{{\star } {\dag }}$; class of movement performed$^{\star }$ (visual feedback)	HPF (1st ord, 16 Hz), LPF (2nd ord, 300 Hz)	100 ms	STD
[65, 66, 141, 144]	SEMT, SAR, TFL, ADDL, GRAC, VASM, RFEM, VASL, BICFL	Footswitch$^{\star }$, load cell$^{{\star } {\dag }}$	BPF (20–420 Hz)	150 ms	LDA-SVM (MAV, ZCN, WL, SSC, MEC)
[53]	RFEM, BICFL, SEMT, GASM, SOL	2× 6-axis force sensor$^{\star }$, footswitch$^{\star }$, joint encoder$^{\star }$	RMS, BPS (20–500 Hz)	NS	SVM (NS)
[85]	VASM, SEMT, ADDL, TFL	2× IMU$^{\star }$	NS	200 ms	Hidden Markov model (MAV, WL, ZC, SSC)
[15, 110]	VASM, ADDL, TFL, SEMT	MoCap system$^{\star }$	NS	NS	SVM (MAV, VAR, MDF, MPF)
[32, 33, 143]	SAR, RFEM, VASL, VASM, BICFL, BICFS, SEMT, TFL, ADDL, GRA	6-axis load cell$^{{\star } {\dag }}$, 2× IMUs	BPF (20–420 Hz)	150–160 ms	LDA/SVM (MAV, WL, ZCN, SSC, MEC)
[86, 87]	TIBA, GASL, BICF, VASL	Footswitches$^{{\star } {\dag }}$; IMU$^{{\star } {\dag }}$	BPF (4th ord, 20–500 Hz)	100–300 ms	LDA/SVM (MAV, VAR, WL, ZCN, SSC)
[120]	TIBA, PERL, GASL, GASM, VASM, VASL, RFEM, BICFL	Class of movement performed$^{\star }$ (visual feedback)	BPF (20–450 Hz)	250 ms	LDA (MAV, ZCN, SSC, WL, ARC)
[58, 111]	BICF, RFEM, VASL, VASM, SAR, GRAC, ADDL, TFL + HAMS reinnervation	Load cell$^{{\star } {\dag }}$, 2× joint encoders$^{{\star } {\dag }}$, 2× IMU$^{{\star } {\dag }}$	BPF (20–450 Hz)	250 ms	same as [140] + Euristic FSM (MEC)
[59, 113, 114, 139, 140]	SEMT, ADDL, TFL, RFEM, BICFL, SAR, GRAC, VASL, VASM	Load cell$^{{\star } {\dag }}$, 2× joint encoders$^{{\star } {\dag }}$, 2× IMU$^{{\star } {\dag }}$	BPF (20–450 Hz)	250–300 ms	LDA/DBN (MAV, WL, ZCN, SSC, 6ord ARC, MEC)
[17]	GASM, TIBA	Load cell$^{{\star } {\dag }}$, joint angle encoder$^{{\star } {\dag }}$, 2× IMU$^{{\star } {\dag }}$	PCA	200 ms	LDA (MAV)
[106]	GASL/SOL	NP	NI	NS	Thresholding (Raw Sign.)
[54, 55]	FIBL, BICF	NP	BPF (4th ord, 20–500 Hz)	256 ms	LDA/SVM/NN (38 mixed domain features)
[6]	GASL, SOL, TIBA	Joint angle$^{{\star } {\dag }}$ (visual feedback)	LPF (7th ord, 5 Hz), NORM	$\sim$ 1 ms	NA (ENV)
[61]	VASL, BICFL	NP	BPF (20–450 Hz), RECT, LPF (5/10 Hz), NORM	NS	NA (ENV)
[135, 136]	HAMS, QUAD	Load sensors$^{{\star } {\dag }}$, goniometer$^{\star }$	BPF (7th ord, 20–1000 Hz), RECT, LPF (5 Hz)	NS	NA (ENV)
[71, 72, 130]	GASM, TIBA	Joint encoder$^{{\star } {\dag }}$, torque sensor$^{{\star } {\dag }}$, 6× load cells$^{{\star } {\dag }}$	HPF(4th ord, 80 Hz), RECT, AVR	150–200 ms	NA (ENV)
[19]	VASM, VASL, RFEM, BICF, SEMT	MoCap system$^{\star }$, sensorized treadmill$^{\star }$, 2× IMU$^{{\star } {\dag }}$	BPF (2nd ord, 30–300 Hz), RECT, LPF (2nd ord, 6 Hz), NORM	300 ms	NA (ENV)

Fields include: paper reference; muscles (input EMG muscle signals employed by the controller); additional sensors and feedback (additional sensor signals employed by the controller and possible feedback provided to the user); EMG signal processing (the sequential processing applied to the input EMG signals; in case of filters, order and cut-off frequencies are included); window sampling (the window length used for the processing and features extraction); classifier (classifier types used on the processed signals and features)

*NP* not present, *NI* not implemented, *NS* not specified, *NA* not applicable, *GRAC* gracilis, *HAMS* hamstring muscles, *QUAD* quadriceps muscles, *VASL* vastus lateralis, *VASM* vastus medialis, *RFEM* rect femoris, *TFL* tensor fasciae latae, *ADDL* adductur longus, *BICFL* biceps femoris long arm, *BICFS* biceps femoris short arm, *SEMM* semimembranosus, *SEMT* semitendineus, *SAR* sartorius, *GASM* gastrocnemius medialis, *GASML* gastrocnemius lateralis, *SOL* soleus, *TIBA* tibialis anterioris, *FIBL* fibularis longus, *GLMAX* gluteus maximus, *GLMED* gluteus medius, *FLEX* flexors muscles, *EXT* extensors muscles, *BPF* band pass filter, *HPF* high pass filter, *LPF* low pass filter, *RECT* rectification, *NORM* max value normalization, *PCA* principal component analysis, *RMS* root mean squared signal, *LDA* linear discriminant analysis, *NARAX* non-linear autoregressive neural network with exogenous inputs, *QDA* quadratic discriminant analysis, *ANN* artificial neural network, *DBN* dynamic Bayes network, *SVM* super vector machine, *LM* Levenberg-Marquardt, *EBA* entropy-based adaptation, *LIFT* learning from testing data adaptation, *ENV* envelope, *IDE* integral of differential EMG, *MAV* mean absolute value, *STD* standard deviation, *MDF* median frequency, *MF* mean frequency, *MPF* mean power frequency, *ZCN* zero-crossing number, *WL* waveform length, *SSC* sign slope change, *ARC* autoregressive coefficients, *MSAR* mean square of ARC, *TDAR* time-domain and ARC combination, *MEC* features from mechanical sensors, *HIST* histogram bin values, *VAR* variance

$^{\star }$Used during calibration phase

$^{\dag }$Used during testing phase

Assuming that a robotic prosthesis can be fastened to the user stump and replace the missing limb, it may be possible to embed the EMG acquisition system on that device. Surface EMG activity is characterized by a high temporal resolution, which makes it practical for the control of an external device. However, signal non-stationarity, motion artifacts, electrode-skin conductivity variations, and channel cross-talk require computationally-demanding signal processing techniques [74]. In order to mitigate these problems, additional mechanical sensors, such as load cells (see also Fig. 3), can be introduced to strengthen the reliability of the control [66]. The sensors listed in Table 3 refer only to those used for the actual control loop: either as input for the device’s high-level control or for its calibration.

Even when using extra sensors, proper muscle choice and EMG processing (e.g. filtering) are essential to guarantee the robustness of the controller.

In order to decode the user’s movement intention, a layer of feature extraction and classification can be added to the control system. Useful features for EMG control are well described in literature [74]. Nonetheless, the choice of the sampling window length is often tailored to the specific processing choices. In fact, the size of the window has to be as large as possible to guarantee the stability of the extracted features, but at the same time—due to signal non-stationarity—the length is calibrated to meet the quasi-stationarity condition.

### Performance assessment

Results concerning control validation and reliability are listed in Table 4, which collects the main findings presented in the selected references.

**Table 4:** Overview of the evaluation measurements and related results

Ref.	Perform-based measurements	Biomechanical measurements	Averaged results	Control delay	Subjects number	Reported limitations
[64]	QA on locomotion performance	NI	NI	50–100 ms	> 1 TFA	NS
[29]	Cadence, Swing and Stance duration	Joint angles and moments, maximum knee flexion	Results in figures only	NS	1 TFA	Sensitive to movement artifacts
[24–27]	Error events analysis	Joint angle NRME, CC	Max error events amplitude = 42 (11 SD); NRME < 6.56 (1.85 SD)%; CC = 0.59 (0.9 SD)	NS	4 ABS	High maximum error amplitudes
[56]	Joint flexion/extension CA	Joint angle RMSE	CA = 92 (7 SD)%; RMSE = 6.2 (0.71 SD)$^{\circ }$	NS	2 TFA, 1 BTFA	NS
[14, 23, 62, 63]	$t_{STRIDE}$; $v_{WALKING}$	Joint angle and joint stiffness; RMSE in joint angle trajectory tracking	$t_{STRIDE}$ = 1.96s; $v_{WALKING}$ = 2.9 km/h; able-bodied resemblance joint trajectories (figures only); RMSE statistical different only with haptic feedback and no-visual	1 ms	1 TFA; 2 ABS	Not appropriate swing control; lack of somatosensory feedback; sensitive to movement artifacts and skin perspiration
[57]	Motor task CA, MCT and MCP	NI	CA = 90.7 (5.0 SD)%; MCT = 1.26 (0.1 SD)s; CP = 96.3 (4.3 SD)%	< 700 ms	6 TFA	Sensitive to electrode shifts and impedance; extensive training necessary
[37]	NI	VAF, RMSE joint angle	VAF > 83%; RMSE < 5.4 (1.2 SD)$^{\circ }$	− 100 ms	3 TTA	Performance being tested only in constant velocity walking task
[16]	MCT	NI	MCT = 1.9 s	NS	5 ABS	Position controller unsatisfactorily during stance phase
[67, 68]	NP	Joint peak power and work respect to state-base controller	Statistical difference of evaluated parameters only with visual feedback (p-val = 0.02)	33 ms	5 TTA	Short training session, experienced high muscular fatigue
[76]	NI	Joint angle trajectories	Results in figures only	NS	10 ABS	No walking speed adaptation, no real-time
[11]	NI	Joint trajectory r-value and SNR	r-value = 0.64 (0.22SD); SNR = 7.42 (2.88SD)	3.3 ms	6 ABS	Position controller unsatisfactorily for limb dynamics
[39, 40]	Number of falling	Joint angle RMSE, EMG contraction level, mean joint torque during balance task	Falling events and applied torque decrease with training; final RMSE = 0.19 (8.78)$^{\circ }$; no significant changes in muscle activation with training	10 ms	6 ABS; 6 TTA	Small sample population; study used visual feedback
[98]	NI	QA of EMG signals and joint angle	Figures only	NS	1 TFA	Sensitive to movement artifacts; sensitive to muscular mass changes
[70]	Locomotion classification accuracy	QA of ankle joint position and shank angular orientation	CA = 86.53 (8.5 SD)%; biomechanical measurements (figures only)	NS	> 1 ABS, > 1 TFA	NS
[5]	Stance time; gait symmetry	Toe-off angle; peak torque; joint trajectories	Qualitatively similar to biological ankle trajectories (figures only)	NS	1 BTTA	Asymmetry on knee flexion during late stance
[65, 66, 141, 144]	Locomotion and transitions CA	NI	Locomotion CA = 91.79–100%; transition CA = 100%	12 ms	5 TFA	Sensor fusion and sound leg instrumentation is necessary to increase accuracy
[53]	Locomotion CA	NI	CA $\approx$ 80–100% (figures only)	NS	5 ABS	NS
[85]	Locomotion CA	NI	CA = 91.46%	NS	100 ms	NS
[15, 110]	Locomotion CA	NI	CA = 91.23%	NS	3 ABS	Tested only healthy subjects
[32, 33, 143]	Locomotion and transitions CA	NI	Locomotion CA $\approx$ 98%; transitions CA > 99%	< 45.2 ms	4 TFA	Real-time test only on non-powered prosthesis; mechanical sensor feature are necessary
[86, 87]	Locomotion CA	NI	CA = 97.9 (1.39 SD)%	NS	5 ABS, 5 TFA	Only limited number of locomotions; major misclassification during gait transitions
[120]	Joint DoF motion CA	NI	1-DoF CA = 93.3 (0.5 SD)%; 3-DoF CA = 84.4 (0.8 SD)%;	50ms	5 ABS, 12 TTA	Best results only combining both tibia and thigh muscles
[58, 111]	Locomotion CA, NWB CA, falls occurrences	NI	with TMR: locomotion CA = 8.9%, NWB CA = 91.0 (4.7SD)%, falls occurrence = 0%; no-TMR: locomotion CA = 10.2%, NWB CA = 86.8 (3.0SD)%, falls occurrence = 2%	NS	4 TFA, 1 TMR	Control degradation over time due to fatigue, electrode shift and skin perspiration; necessity of mechanical sensors for high accuracy; small number of subjects
[59, 113, 114, 139, 140]	Locomotion and transitional CA, effects on classification errors	NI	Locomotion CA < 99%; transition CA = 87%; classification errors during stairs were more disruptive	< 20 ms	7 TFA	Control degradation over time due to fatigue, electrode shift and skin perspiration; testing is performed in only one sessions
[17]	Questioner on control comfort	Inclination CA	CA > 95%; comfort higher when no classification error (accepted error $\le 5^\circ$)	NS	2 TFA	Experienced high muscular fatigue; lack of sensory feedback
[106]	NI	QA of EMG signals	Results in figures only	NS	1 ABS	Not tested on amputee; only walking activity control
[54, 55]	Locomotion CA	NI	CA = 99.06 (0.87SD)%	32 ms	5 ABS	Not tested on-line
[6]	NI	Joint angle trajectory frequency content	mean frequency = 5.4 (0.3 SD) Hz, qualitatively similar to biological ankle	NS	1 TTA	Temporal variation of the EMG signals are not accounted for
[61]	Subject qualitative report	QA of knee joint position and torque	Control interface did not feel natural; able-bodied resemblance joint trajectories (figures only)	NS	1 ABS with ABA	Experimental tuning of the parameters is necessary
[135, 136]	QA of joint trajectories with respect to ABS	Joint angle trajectory RMSE	Results in figures only	NS	1 ABS with ABA	Model parameters are tuned manually; control instable with biarticular muscles
[71, 72, 130]	NI	Toe-off angle, joint net work, peak power, joint torque vs angle	Net work performed higher than the biological norms; not substantial difference in joint moments between intrinsic controller and EMG-driven	2 ms	1 BTTA; 3 TTA, 3 ABS	Further studies on the real metabolic cost benefits are required
[19]	NI	Joint torque NRMSD with respect ABS	NRMSD $\le$ 0.24 (0.11 SD)	< 50 ms	1 ABS	Complex subject-specific calibration; required validation on more subjects and hardware

Fields include: paper reference; performance-based measurements (e.g. cadence, stance/swing time, stumble rates, etc.); biomechanical measurements (e.g. joint trajectory deviations, peak angles, net work, etc.); averaged results (averaged or worst subject-case results); control delay (time required for the generation of the high-level control output from the acquired relevant signals, including processing); subjects (number of subjects); reported limitations (limitations reported by the authors)

*NP *not present, *NI *not implemented, *NS *not specified, *QA *qualitative analysis, *CA *classification accuracy, *RMSE *root mean squared error, *NRMSD *normalized root mean squared deviation, $t_{STRIDE}$ time stride, $v_{WALKING}$ walking self selected velocity, *VAF *variance accounted for, *MCT *motion completion time, *MCP *motion compilation percentage, *DoF *Degree of Freedom, *TFA *transfemoral amputee, *BTFA *bilateral transfemoral amputee, *TTA *transtibial amputee, *BTTA *bilateral transtibial amputee, *ABS *able-bodied subjects, *ABA *able-body adaptor

The efficacy of an EMG-driven control framework is always a trade-off between intuitiveness, system response time and accuracy of movement selection [35]. Consequently, it is challenging to define a threshold of acceptability of a given implemented solution. Bias and variance of the myoelectric control parameters, as well as in the input signals, depend on numerous conditions and they can heavily impact performance [3]. The reviewed manuscripts showed limited amount of evidence on assessing patient safety and security, probably due to their prototypical stages of the development. However, there are studies focusing exclusively on EMG-driven control faults and safety issues, which analyze critical situations for stumbling and falls [145].

The different number of subjects and the average results presented in Table 4 show that a standardized evaluation is missing for defining the overall performances of MLLP EMG-driven controllers, even at the prototype stage.

During this review, the authors found a strong correlation between the type of evaluation and the type of implemented neuro-control. In fact, while IEC manuscripts focus their analyses on biomechanical measurements, CIC controllers focus mainly on evaluating performance-based measurements.

An analysis of the literature also shows that control delay is an important parameter to evaluate the controller performance in real scenarios [137]. In particular, to guarantee control stability while considering the HITL factor, the total maximum control delay between the signal window sampling and the actual output value has to be less than the human physiological electromechanical delay $d_\text {EM}$. The human $d_\text {EM}$ is caused by the time that a neural signal requires to generate the electrical depolarization of the muscle tissue, to consequently result in a mechanical force, and finally joint displacement. The delay has been estimated to be $d_\text {EM} \approx 100$ to ${150}\;{\text {ms}}$ for the lower limb [123, 129]. If the generation of the prosthetic mechanical movement requires longer time than $d_\text {EM}$, the prosthesis will be always delayed with respect to the volition of the user, which limits the usability of the controller and may generate risky conditions. Even though the prosthesis’ motion generation delay is a crucial metric to evaluate the EMG-driven control, close to half of the reviewed manuscripts do not provide such information. Long delays are especially problematic if they are prone to a logical deadlock, where the system comes to a halt because its subsystems (human and the prosthesis) are waiting for each other to take action. For example, if the controller requires completion of a (partial) step cycle to detect a user-intended switch from walking to stair climbing, while the user is not at comfort or able to perform the first stair climbing step with the presently selected walking controller, then the switch will never occur.

## Discussions

The results presented in “Results” section can be further discussed to identify patterns, prospects and limitations of the various approaches, to understand how future research and development into EMG-driven controlled MLLPs is most promising.

The structure of the discussion follows that of the results, i.e., uses the same four topics as subsections. Furthermore, this section presents an additional discussion in “Clinical considerations” section, regarding the clinical implications of developing an effective neuro-controller, in particular an EMG-driven controller. This is important to provide a general interpretation of the results in the context of other evidence.

### Neuro-control

High-level controllers for lower limb prostheses, and in general lower limb robotics, aim to restore both rhythmic locomotion (CIC) and volitional movements (IEC). Unfortunately, very little is known about how these two processes cooperate in humans, but it has been proposed that these two paths can bidirectionally interact and cooperate with each other [20]. This lack of knowledge generates a particular dichotomy of EMG-driven control in restoring either rhythmic or volitional movement. CIC and IEC aim exactly in the control of either type of movements (Fig. 4).

Similarly as in human CPGs, prosthetic CIC does not require any conscious human involvement [138]. Rhythmic motor patterns, like walking, compose almost the totality of human basic locomotion, and therefore constitute the most important type of movements to restore in amputees. Rhythmic motor patterns are easily detectable and reproducible, by employing basic concepts of data analysis on prosthesis kinetic and kinematic measurements [100, 127]. For this reason, pattern recognition control techniques are employed for implementing CICs and are the most widely used. In fact, as stated in “EMG-driven working principles” section, pattern recognition uses data from embedded sensors in the prosthetic device (e.g. load/pressure cells, foot switch, joint encoders and IMU) to identify the locomotion type and change the control law parameters accordingly. The introduction of EMGs and therefore the integration of user volition information in this control paradigm has been investigated mainly with two objectives: using the myoelectric signals as additional information to increment the number of classes of movements that can be controlled, and boosting their recognition accuracy. These controllers therefore inherited the same controller capabilities of their non-EMG MLLPs predecessors: high reliability during rhythmic locomotions in spite of their lack of intuitive control. Therefore, CICs are the most effective option when the main objective of the MLLPs is to restore walking, which is the case for a major target group of lower limb prosthesis users. In contrast to rhythmic movements, volitional motions are not characterized by phase-dependent trajectories, nor by correlated conditions between repetitive movements. Due to the redundancy in the musculoskeletal system, identical limb configurations can be generated from different muscle activations [77]. This particular property of the human musculoskeletal system makes it significantly more difficult to design a controller that is able to obtain a reliable solution through the processing of only EMG signals for the voluntary control of the artificial joint. Direct and model-based controllers in IECs tackle this issue in two different ways: the former employs signal processing to reduce these aforementioned redundancies, while the latter uses modeling in order to characterize them. Despite this technological difficulty, users can employ this type of control to voluntarily and continuously modulate joint flexion-extension with higher degree of freedom respect to the previous class of controllers.

Collected results showed that only two research groups attempted to unify CIC and IEC in a unique control strategy [72, 113]. The first research was conducted at the former Center for Bionic Medicine, Rehabilitation Institute of Chicago [58, 111]. They adopted a pattern recognition control algorithm, in combination with Targeted Muscle Reinnervation (TMR), to implement a hybrid CIC-IEC. Depending on the device state, the FSM could decide either to control rhythmical locomotion or, in case of a non-bearing motor task, give the user the freedom to control the joint voluntarily.

Another exception to the CIC/IEC dichotomy can be seen in the work conducted at the MIT Media Lab [71, 72, 130]. In their studies, the EMG signal proportionally regulates the gain of a Hill-type muscle model to generate additional plantar-flexion force. Deviation from the intrinsic controller output was enabled depending on the type of locomotion performed and the level of muscular activation: this was especially used to generate an additional push-off when required by the user. Their approach employed standard pattern recognition techniques for locomotion control, while applying direct voluntary or model-based techniques to deviate from such intrinsic control during particular locomotive states. These studies recognized the strengths of the two strategies and attempted to simultaneously preserve both high reliability and seamless control.

This particular choice on the type of neuro-control and the type of movement to be addressed has a direct consequence on the type of controller that should be implemented. Their particular working principle is discussed in the following section.

### EMG-driven working principles

Walking represents the most important locomotion in humans: on average 10,000 steps per day for the younger population ($\le 65$ years old) and 7000 for the older ($>65$ years old) [10]. Consequently, SlA and SpA are usually the most implemented, since they are essential to accommodate different walking patterns based on the cadence and terrain conditions. Table 2 displays the additional modalities (locomotion types) that are allowed to be performed from the controllers, apart from walking. While an EMG-driven pattern recognition approach has to declare the modalities that can be controlled specifically in the framework design, direct-control and model-based approaches are able to deliver the control for any type of locomotion. However, even if they theoretically could, there is a lack of experimental validation for this aspect. In fact, some of the reviewed works did not perform any validation regarding such capabilities, as they only report results of ground-level walking [26, 37, 61].

Despite this limitation, the strength of pattern recognition algorithms, such as ML, is the ability of learning and recognizing peculiar features from a given data set. This skill becomes useful for the case of multi-dimensional and complex data streams that have to be analyzed and labeled in real time, such as EMG. Moreover, EMG signals are highly irregular and non-stationary, and amputees could develop abnormal co-contraction features in the residual limb, making techniques of direct control particularly difficult.

Another important characteristic that distinguishes pattern recognition techniques from other control techniques, is the reduced user training time, as can be seen in “EMG-driven working principles” section. Clinical evaluations of myoelectric prostheses have correlated the reduced training time to a higher device acceptance [35]. Even though there are no standards to establish an optimal training time, prolonged and repetitive sessions might result in cognitive and physical exhaustion. However, it has to be noticed that no pattern recognition study explored multiple testing sessions and therefore dealt with recalibration issues. Depending on the robustness of the developed pattern recognition EMG-driven controller, calibration time might be necessary before each session, correspondingly worsening device acceptance.

This discussion has clarified that the choice of the type of the controller affects the type of processing and the type of signals. Further discussion on the type of processing related to the type of EMG-driven working principle is discussed in the next section.

### Neural input and processing

The choice of which muscle signals to use represents a fundamental topic in EMG-driven control. This choice is influenced not only by the architecture of the controller, but additionally by the artificial joint’s mechanical and electronic design, from the amputation level and finally by the surgical muscle reattachment [51]. The latter two points are subject-specific, which leads to user inter-variability regarding choices of the muscle signals and consequently the EMG-driven control technique. Additionally, the biological joints are controlled from a different number of muscles, which limits the number of signals that can be used after amputation depending on the joint. Moreover, the presence of bi-articular muscles—and therefore the kinematic and dynamic coupling between the different joints—may also affect the number of channels that can be used. Finally, MLLPs mechanics and electronics (such as prosthesis weight, volume, and battery-life) can affect the number of sensors that can be embedded.

Related to this issue, the reviewed literature tended to go in two opposite directions. Some of the studies used a small number of EMG-sensors, and placed them on big proximal muscle groups that would still be present after the amputation, as in [6, 24, 29, 56, 61, 63, 64]. Instead, other studies acquired readings from multiple muscles in order to collect redundant information, and to analyse how the controller performance changes by using a variety of signal processing techniques [66, 70, 120]. Whereas the latter requires an initial analysis, this approach can be used to better tailor the controller to user necessities, leading to an improved performance when the number of EMG sensors is higher. This particular choice is usually related to pattern recognition controllers and explains why this class of controllers has usually more EMG channels.

Additional sensors have been extensively used in EMG-driven control in order to improve their accuracy (Fig. 3). In fact, a common strategy is to add mechanical sensors (e.g. IMU and pressure cells) to the prosthetic device to have more reliable information to compensate for the high variability of EMG signals and their sensitivity to noise. For example, using a foot switch for detecting gait events (e.g. heel strike and toe off) is still the main method for the identification of stride phases. This identification can be used additionally to adapt different lower-level control strategies or for disabling/enabling the EMG-driven control with respect to standard approaches [6, 58].

The use of supplementary mechanical sensors (e.g. encoders) in addition to EMG electrodes can also be used for restoring the missing sensory feedback from the amputated limb. It was found that the most common approach was to provide the user the joint angle position during joint movements through visual representation. Indeed, feedback is particularly important in the IEC class of controllers. In this particular case, since the user is in continuous and full control of joint flexion and extension, it is beneficial to return information about the MLLPs configuration. The use of visual feedback, though, is impractical during every-day activities. A potential solution is to employ a haptic feedback, such as vibrotactile stimulus, coding for joint angle position, as suggested by [6, 62]. However, only one work was found to investigate the use of haptic feedback in conjunction with EMG-driven control [14]. Their results suggested that this was mostly beneficial when visual feedback was limited.

Processing EMG signals usually follows two possible directions, which is mainly influenced by the type of neuro-control implemented from the high-level controller. For IEC, the common approach is to extract the fully rectified envelope of the signals around the most informative bandwidth (around 10 to 500 Hz), and then to normalize the relevant signals for their maximum values. Instead, for the case of CIC, where frequency-based features are usually employed, band-pass filtering is applied in order to delete low- and high-frequency noise components.

Classification of EMG features, as shown in Table 3, is usually applied in pattern recognition control. In non- EMG MLLP, the most used are heuristic rule-based classifiers, like FSM impedance controllers [116] or decision tree controllers [142]. By providing the right set of rules, these controllers can identify a gait phase [115] and additionally identify different types of locomotion, such as walking or slope climbing, driving the mid-level impedance control accordingly [117, 118]. The same purpose of heuristic rule-based classifiers can be achieved with automated pattern recognition algorithms through ML. Such classifiers are usually more robust to outliers but they all require a priori offline training, often on data derived directly from the final user. Linear discriminant analysis (LDA) [140], quadratic discriminant analysis (QDA), Gaussian Mixture Models (GMM) [128], Support Vector Machines (SVM) [66] and Artificial Neural Networks (ANN) [5] are examples of possible solutions that have been explored, each with relative merits and drawbacks. Due to these properties, the ML approaches are usually preferred for EMG-driven pattern recognition controllers on neural signals features [32, 53, 65, 66, 86, 120].

This review has not found any use of Deep Learning (DL) applications for EMG-driven control in MLLPs, in contrast to their upper limb counterparts. Note that DL is a subset of ML, characterized by the ability to self-learn meaningful signal characteristics, rather than requiring features to be defined in advance. The choice of features for classification is a challenging process and impacts long-term performance of the EMG-driven controller [125]. For this reason DL classifiers might represent a solution for future implementation aiming in improving state-of-the-art ML performances [22].

In addition, this review shows that ML strategies are not limited to CIC: they can be used as mapping tool for trajectory generations in IECs, such as in [24, 37], or for reducing signals variability [56, 57].

The diversification regarding the type of neuro-control found in literature complicates the comparison of all studies: the presence of multiple control and processing methods makes it difficult to isolate the effects of each choice on the final outcome. Instead, as presented in the following section, the performance validation metrics and their analysis highly depend on the control design choices.

### Performance assessment

The first attempts to introduce EMG signals in the control loop of lower limb prostheses were made in the 70’s with the work of Horn [64] and Donath [29]. Although myoelectric controllers have evolved in the last 40 years, current solutions are still far from being integrated into lower limb prosthetic commercial devices. The main obstacle to this is represented by safety and risk assessment after EMG is introduced in the control loop.

Consistently with the available literature on myoelectric controllers, the self-reported major limitations of EMG-driven controllers for MLLPs are related to instabilities generated from high sensitivities to EMG noise, movement artifacts, and electrode impedance, usually due to skin perspiration. Additionally, pattern recognition approaches mainly report limitations about the fact that only a limited number of locomotion types or motor tasks can be controlled, as already discussed in “EMG-driven working principles” section. The main impediment to model-based control frameworks is represented by the manual tuning of parameters during experimentation, which is crucial for acceptable control. Instead, in direct control, only one manuscript reported that extensive subject training was necessary to compensate for the increased cognitive load [62]. In fact, users required constant awareness about the prosthesis configuration, since the extension and flexion of the knee joint were completely volitional.

Aside from the specific control implementations, the ultimate purpose of a prosthesis is to operate synergistically with the human body and replicate a behaviour that is as similar as possible to the missing biological limb. Therefore, evaluating the ability to generate human-like joint trajectories and forces during locomotion represents an important indication of the performance of the control. Additionally, investigation of performance-based measures, such as changes in cadence, stride length, walking speed, and their application to standard prosthesis controllers represents a strong validation of the performance with respect to the state-of-the-art.

As reported in “Performance assessment” section, these two types of investigations were usually not conducted simultaneously. Depending on the type of neuro-control, either performance-based or biomechanical measurements are used for performance analysis. This particular finding makes it difficult for this review to assess a generic comparison between controllers based on their reported findings, since no common accuracy and reliability metrics could be found.

An additional challenge was found when attempting to compare MLLPs performances with able-bodied kinematics and dynamics, because the results were often qualitative or limited in terms of statistical analysis. [76, 106, 136]. CIC controllers—while usually providing more accurate quantitative analyses with respect to IEC—are usually only limited to classification accuracy of motor task and the type of locomotion recognition. Although it is a solid parameter for the evaluation of FSM controllers, it does not provide a clear measure of the system capability. Few works instead, compared the accuracy of their EMG-driven approach with respect to standard or commercially available CIC [59, 113]. Only few other manuscripts proposed analytical correlations between (1) the recorded accuracy, (2) the final performance of the controller, and (3) the errors in the classification process that could lead to a dangerous failure [59, 145].

Additionally, related to this problem, this review has identified critical lack of attention to significant measurements, such as the control delay. While this parameter is not a direct measurement of the high-level control, it is of importance for the evaluation of the whole HRI.

These issues refer to the concept of bench-marking: the definition of guidelines to be followed in the iterative process of design and development of a new generation of MLLPs. While accredited guidelines are available for testing exoskeletons [91] and bipedal robots [122], standardized experimental methods for evaluating MLLPs are lacking [48]. With this purpose in mind, recent reviews have categorized the evaluation on MLLPs in three methodologies: patient-reported outcomes, performance-based measures and biomechanical measures [42, 48]. Moreover, these reviews highlight the discrepancy between the evaluation metrics used for research and commercial MLLPs, which focus instead on questionnaires on comfort, sense of security and quality of life [101]. This trend can be explained due to the fact that the commercial market is more focused on end-user satisfaction for marketing purposes, whereas researchers tend to focus on engineering performance metrics.

Before arriving to final conclusions, it is necessary to contextualize the obtained results in the clinical situation of lower limb amputations. This discussion is critical to understand the real necessities in this health sector and how myoelectric MLLPs can constructively collaborate to address them.

### Clinical considerations

The literature depicts a particularly serious perspective for lower limb amputations and prosthesis utilization. Despite the improvement of medical treatments, the lower limb amputation rate has not substantially changed [38, 105]. This is due to the fact that the population at-risk for lower limb amputation (elderly and diabetic patients) is constantly increasing. In fact, vascular related diseases remain the leading cause for lower limb amputations in industrialized countries [28, 49, 90, 121].

Considering clinical aspects, it is important to note that critical psychosomatic conditions and secondary injuries can emerge over time in amputees. Indeed, evidence suggests that both phantom limb pain and joint degeneration due to compensatory movements can be avoided with neural interfaces and powered prostheses [78, 96, 134].

Moreover, relatively high prosthesis abandonment is influenced by the acceptance rate of lower limb prostheses, which are associated with user perception of inadequate controllability of the device, specifically a lack of intuitiveness in the control [4]. In addition to deficiencies that result from aforementioned mechanical challenges (e.g. noise, heaviness, battery life), the lack of intuitive control likely contributes to the relatively low popularity of powered prostheses. This aspect advocates for the discrepancy between research and the commercial prostheses reported in “Performance assessment” section, where patients’ reports play a major role with respect to the performance and biomechanical capability of the device.

Therefore, implementing volitional control can potentially result in large improvements in the usability of these prostheses. In fact, MLLPs can already recognize and control most of the locomotion modes. However, important classes of motor tasks involving the knee and ankle have been omitted from this set of movements, making it difficult or impossible for amputees to perform them [56]. For example, body shifting position while sitting or particular joint flexion and extension motions are essential during daily activities, such as donning and doffing a shoe, or entering a car. These movements can be executed only using a prosthesis with neural-driven control, unless the repositioning of the joints must be done by hand. Thus, the implementation of this class of movements and the increase of the number of activities in powered prostheses would augment user freedom of motion in daily life. This is the key in making the prosthesis become part of the user routine, which will increase the feeling of embodiment, and will lower the probability to abandon the prosthesis [13].

However, the results of this review have shown several drawbacks (e.g., subject-specificity and high noise sensitivity of EMG signals) related to the adoption of EMG-driven control for MLLPs and no solid representative emerged as the predominant solution. Therefore, the adoption of such technology still remains disputable for MLLPs. Nonetheless, it is important to consider that novel invasive technologies grant a direct connection with the neuromusculoskeletal system (e.g., osteointegration, TMR, implantable EMG sensors). These techniques have been clinically tested in humans and demonstrated the potential to attenuate such limitations of standard myoelectric MLLPs [36]. In summary, it should be possible to substantially improve the performance of neuro-controlled MLLPs by creating a more reliable connection between the robotic limb and the human.

## Conclusions

This review has assessed the current state-of-the-art in EMG-driven control methods for MLLPs in the attempt to identify their prospects and limitations, and to formulate suggestions on future research and development. Four major topics of investigation were addressed: neuro-control of the device, EMG-driven working principles, neural input and processing, and performance assessment.

The authors found an evident lack of quantitative and standardized measures regarding sensitivity and risk analysis. Additionally, this review has not found evidence of meaningful comparisons of EMG-driven control in MLLPs with respect to standard adaptive control. Likewise, no comparison was found with commercial products. Moreover, due to missing guidelines in MLLPs development and evaluation, performances metrics are tailored to the type of the implemented neuro-controller. This issue complicates the execution of a quantitative analysis between the reviewed EMG-driven controllers.

However, the reviewed literature also suggests that there are certain preferences regarding control principles depending on the type of movement intended to be restored. If the interest is to provide the prosthesis control during rhythmical locomotion, pattern recognition controllers are to be preferred. This solution is suggested for non-active people that use the prosthesis only for simple daily tasks (e.g., walking, sitting, ascending and descending stairs). For these tasks, this type of controller guarantees a lower cognitive load and more stability. In contrast, control of volitional movements is preferred with direct and model-based controllers. This approach lets amputees control their prosthesis during complex tasks with higher freedom and autonomy, at the expense of higher cognitive effort and attention.

Despite the increasing interest in EMG-driven controllers for MLLPs, a reliable and effective approach that can be introduced into powered prostheses has not yet been formulated. Particularly, continuous developments in ML have resulted in a persisting focus on pattern recognition techniques, which despite allowing for high stability and easier integration, remain a risky approach as they have not proven to be able to tackle the inadequate user control yet. In addition, the full potential of using neural signals is left unexploited, such as targeting direct and voluntary manipulation of the prosthetic joint, which would give the user complete freedom of movement. Based on the evidence reported here, the authors believe that introducing a reliable and effective control, able to integrate both rhythmic (CIC) and volitional (IEC) motor tasks, will promote the use of powered MLLPs despite their current limitations. First attempts in this direction have been investigated in this review. They have demonstrated the possibility to exploit both the potentialities of the two neuro-controllers.

The real efficacy of EMG-driven controllers have yet to be clinically validated. Furthermore, EMG-driven controllers still have to overcome inherent drawbacks such as high noise sensitivity of EMG signals, elevated inter- and intra- subject signals variability, and a difficult integration of electrodes in the socket. However, current advancements in invasive solutions for bionic prostheses have demonstrated to strengthen the connection of the HRI, creating a more reliable neuro-control and attenuating the aforementioned limitations. Therefore, it is likely that the combination of novel invasive interfaces, more advanced decoding algorithms (e.g., DL or modeling) and seamless EMG-driven control will eventually promote the use of powered MLLPs. This claim is strongly related to clinical evidences about the necessity to provide amputees with powered support to avoid psychosomatic conditions and secondary degenerative musculoskeletal pathologies.

Eventually, neural-driven controllers will acquire the capability of closing the sensori-motor loop. Users will be provided with sensory information about touch and proprioception, through the prosthesis, to modulate the control in closed-loop, allowing bioengineers to get closer to the goal of giving back what amputees have lost.

## Data Availability

Not applicable.

## Abbreviations

ANN: Artificial Neural Networks
CIC: Computational Intrinsic Control
CNS: Central Nervous System
CPGs: Central-Pattern Generators
DL: Deep Learning
EMG: Electromyography
FSM: Finite-state machine
GMM: Gaussian Mixture Models
HITL: Human-in-the-loop
HRI: Human–Robot Interaction
IEC: Interactive Extrinsic Control
IMU: Inertial Measurement Units
LDA: Linear discriminant analysis
ML: Machine learning
MLLPs: Microprocessor-controlled lower limb prostheses
MS: Musculoskeletal
QDA: Quadratic discriminant analysis
SlA: Slope adaptation
SpA: Speed adaptation
SVM: Support Vector Machines
TMR: Targeted Muscle Reinnervation
